# The Tumor Plastic Surgery Technology versus Traditional Repair Technology on the Repair of Large-Area Skin Defects after Maxillofacial Tumor Resection: A Randomized Controlled Trial

**DOI:** 10.1155/2022/3004695

**Published:** 2022-05-26

**Authors:** Xue-Feng Zhang, Zheng Chu, Hong-Li Yang, Bing Zhao

**Affiliations:** ^1^Department of Orthopedics, Panzhihua Iron and Steel Group General Hospital, Panzhihua, Sichuan Province, China; ^2^Department of Neurosurgery, Panzhihua Iron and Steel Group General Hospital, Panzhihua, Sichuan Province, China; ^3^Department of Science and Education, General Hospital of Pangang Group, Panzhihua, Sichuan Province, China; ^4^Plastic Surgery Department of Chengdu Fuli Plastic Surgery Hospital, Chengdu, Sichuan Province, China

## Abstract

**Objective:**

To explore the effect of tumor plastic surgery on the repair of large-area skin defects after maxillofacial tumor resection.

**Methods:**

90 patients undergoing maxillofacial tumor resection in our hospital from March 2019 to March 2020 were selected and randomized 1 : 1 to receive either tumor plastic surgery (experimental group) or traditional repair (control group). The clinical efficacy and facial cosmetic improvement of the two groups were compared. The Patient and Observer Scar Assessment Scale (POSAS) was used to evaluate the surgical outcomes of the two groups, the Profile of Mood States (POMS) was used to evaluate the patients' psychological status, and the Generic Quality of Life Inventory-74 (GQOLI-74) was used to assess the quality of life of patients.

**Results:**

Total clinical effective rate of the experimental group was significantly higher than that of the control group (*p* < 0.001). A higher excellent rate of facial cosmetic improvement was observed in the experimental group versus the control group (*p* < 0.001). Significantly lower POSAS scores of the experimental group than the control group were observed (*p* < 0.001). The POMS scores of the experimental group after treatment were lower than those of the control group (*p* < 0.001). Tumor plastic surgery resulted in a remarkably higher GQOLI-74 score in the patients versus traditional repair (*p* < 0.001).

**Conclusion:**

Tumor plastic surgery is a promising alternative for patients undergoing maxillofacial tumor resection. It can effectively promote the recovery of facial morphology and physiological function of patients, with high clinical efficacy, so it merits promotion and application.

## 1. Introduction

Maxillofacial tumors are tumorous lesions in the oral and maxillofacial regions, including benign tumors, malignant tumors, and tumor-like lesions [1–3]. Benign tumors comprise mixed tumors of the parotid gland and schwannomas of the neck in the salivary glands, and malignant tumors are mainly oral squamous cell carcinomas, such as palate cancer, tongue cancer, and buccal cancer [4]. The early clinical manifestations of the disease are local ulcers, and it infiltrates the surrounding and deep tissues with progression, leading to pain and facial movement dysfunction, compromising the patient's quality of life. At present, the mainstay of treatment for maxillofacial tumors is surgery, yet it would cause massive loss of facial and neck tissues, resulting in impaired language ability and chewing function [5]. Moreover, the removal of maxillofacial tumors has aesthetic implications, resulting in an unsatisfactory cosmetic outcome and a negative attitude toward social activities. Therefore, the maximum restoration of the patient's facial cosmetic appearance remains a key clinical issue to be addressed [6, 7]. Despite the current widespread utilization, conventional surgical repair treatment presents inferior performance with respect to functionality. Fortunately, tumor plastic surgery has been proven to be a mature and effective technique in breast cancer [8, 9]. Nevertheless, no study has yet specifically explored the outcome of tumor plastic surgery for large-area skin defects after maxillofacial tumor resection, to our knowledge. Maxillofacial tumors are formed due to hyperplastic changes within the skin and its underlying tissues caused by various factors. Conventional surgical repair is to suture the skin after tumor removal, but postoperative scarring and indentation may occur, compromising the patient's postoperative appearance and oral function. Currently, studies have shown that squamous cell carcinoma and basal cell carcinoma are the most common malignant tumors in the maxilla. Such malignant tumors need extended resection surgery, and there are various methods to repair the soft-tissue defect after surgery, and the repair of the defective soft tissues requires complete coverage of the trauma while also ensuring effective restoration of facial function. Accordingly, the present study was conducted to assess the effects of tumor plastic surgery by recruiting ninety patients with maxillofacial tumor resection admitted to our hospital from March 2019 to March 2020.

## 2. Study Design and Participants

### 2.1. Participants

A total of 90 maxillofacial tumor resection subjects admitted to our hospital from March 2019 to March 2020 were recruited and randomized 1 : 1 to the experimental and control groups according to the order of admission.

### 2.2. Eligibility and Screening

Participants were included per the following: (1) confirmed as the maxillofacial tumor by imaging examination and pathologically diagnosed by the preoperative biopsy of the tumor tissue specimens; (2) clinical stage II-III; (3) treated with surgical resection, and there were no surgical contraindications; (4) maximum diameter of facial defect of <10 cm; (5) aged ≤66 years; (6) with complete clinical data; (7) undersigned informed consent form was obtained from all patients; and (8)this study was conducted in accordance with *Helsinki Declaration* [10].

Patients were excluded if they (1) received radiotherapy simultaneously; (2) had coagulation dysfunction; (3) had other oral diseases; (4) had severe liver and kidney dysfunction; (5) had malignant tumors, severe cardiovascular and cerebrovascular diseases, or cardiopulmonary insufficiency; and (6) could not cooperate with treatment due to mental illness.

This study was reviewed and approved by the ethics committee of Panzhihua Iron and Steel Group General Hospital (approval no. 2018-29802).

### 2.3. Interventions

Both groups of skin tumors were surgically removed with the aid of a microscope. With the patient in a supine position, the patients were given general anesthesia, and surgical operations were performed by the same medical team. The tumor was excised using Mohs' microscopic technique (a combination of cosmetic dermatologic surgery techniques and special frozen tissue sectioning, where the boundaries and depth of the tumor lesion were determined by histology), and a vascularized forearm flap repair and a local rotational flap repair were performed after complete resection of the tumor and surrounding tissue.

The control group received traditional repair techniques to design the shape and size of the skin flap according to the patient's facial defect. The operation area with methylene blue was marked, the skin from the distal end of the donor area was cut, and the subcutaneous tissue was separated to the surface of the pectoralis major muscle. The free skin flap was passivated to perform a subcutaneous tunnel from the pectoralis major to the neck, and the flap was turned over and pulled to the facial defect through the subcutaneous tunnel. The blood vessel was anastomosed, and the edge of the skin flap was mattress-sutured, a drainage strip was placed, and the donor area wound was sutured.

The experimental group used tumor plastic surgery technology. The blood supply of the wound surface and tear tissue was observed, skin tissue was preserved to the greatest extent, and hydrogen peroxide was utilized to disinfect the deep tissues of the wound if the maxillofacial tissue was ischemic or free and was connected to a small part of the pedicle tissue. A layered suture was used to suture the deep subcutaneous tissues, attention should be paid to the texture of the skin, and the forced suture was prohibited during the suturing process. When the wounds with tissue defects were repaired, full consideration should be given to factors such as the contour lines between vital facial organs, dermatoglyphic direction, and morphology. For areas with greater facial tension such as the nose and forehead, double-leaf rotating flaps were used. For wounds with loose soft tissues on the cheeks and ears, rhomboid skin flaps were used. In cosmetic repair, forced suturing was prohibited. The displacement and eversion of mouth, eyes, nose, and other parts were observed during the suturing process, and rotation flap and advanced skin flap were utilized for small defect areas. After the facial trauma was repaired, it was sutured in layers from the inside to the outside to ensure that the sutures between the skin fissures were tension-free and the sutures between the skin incisions were flat, to avoid overlapping and shrinking.

### 2.4. Outcome Measures

The clinical efficacy is classified as markedly effective if the patient's mouth opening and oral occlusal relationship return to normal, there is no scar in appearance, and the alignment is good. The clinical efficacy is categorized as effective if the patient's mouth openness and oral occlusal relationship basically return to normal, and facial scars are not obvious. The clinical efficacy is considered ineffective if the patient's oral occlusal relationship does not return to normal, and the language function and occlusal are affected. The total clinical effective rate = (markedly effective + effective)/total number of cases × 100%.

The high-resolution digital camera (model: Canon PowerShot G7 X Mark II) was employed to shoot the photos of facial cosmetic improvement of the two groups of patients before and after the operation, with consistent posture and exposure. The improvement of the patient's face was evaluated by two skilled doctors with more than 5-year seniority in facial plastic surgery. The score ranges from 0 to 10 points. Excellent improvement is rated as ≥8 points, good as 6-<8 points, moderate as 4-<6 points, and poor as <4 points. The excellent rate = (excellent + good)/total number of cases × 100%.

The Patient and Observer Scar Assessment Scale (POSAS) [11] consisting of patient scar assessment scores (PSAS) and the observer scar assessment scale (OSAS) was used to assess the scar. PSAS was used for self-evaluation on the dimensions of the degree of pain or itching, color, hardness, thickness, and flatness of the operation area. Each item ranges from 1 to 10 points, with a full score of 70 points. The lower the score, the better the condition. OSAS was applied to evaluate the degree of hyperemia, color, thickness, softness, and compliance of the patient's surgical area. Each item is scored 1-10 points, with a total score of 70 points. A lower score indicates a more satisfactory result of the surgery.

The profile of mood states (POMS) [12] consisting of 40 items was applied to assess the mood state of the two groups after treatment. The score is comprised of negative emotion scores and positive emotion scores. The lower the score, the better the mood.

The Generic Quality of Life Inventory-74 (GQOLI-74) [13] was used to evaluate the quality of life of the two groups of patients after treatment. The scale is scored on four dimensions of mental function, physical function, social function, and material life state, with a total score of 100 points. A higher score suggests a better quality of life.

### 2.5. Statistical Analyses

The statistical analysis was done by SPSS21.0 software, and the graphics were visualized by GraphPad Prism 7 (GraphPad Software, San Diego, USA). The enumeration data including clinical efficacy and facial improvement were represented as [*n* (%)] and examined via a chi-square test. Measurement data including POSAS scores, POMS scores, and GQOLI-74 scores were expressed as (*x* ± *s*) and compared using a *t-*test. For all the tests, statistical significance was set at *p* < .05 (two-tailed).

## 3. Results

### 3.1. Comparison of Baseline Data

The baseline characteristics were similar between the two groups in gender ratio, average age, BMI value, tumor type, pathological stage, education level, occupation, and living conditions (*p* > 0.05); see Table 1.

### 3.2. Comparison of Clinical Efficacy

The total clinical effective rate of the experimental group was significantly higher than that of the control group (*p* < 0.001, Table 2).

### 3.3. Comparison of Postoperative Facial Improvement

Table 3 reported a higher excellent rate of facial improvement in the experimental group versus the control group (*p* < 0.001).

### 3.4. Comparison of POSAS Scores between the Two Groups

Significantly lower POSAS scores of the experimental group than the control group were observed (*p* < 0.001), as listed in Table 4.

### 3.5. Comparison of POMS Scores between the Two Groups

**Figure 1:**
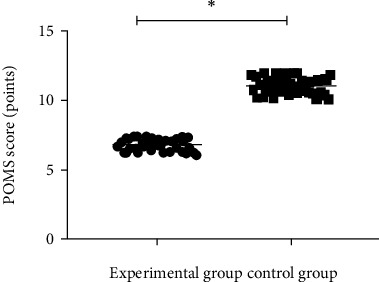
Comparison of POMS scores between the two groups after intervention ($\overset{¯}{x}\pm s$). Note: the abscissa represents the experimental group and the control group, respectively, and the ordinate represents the POMS score, points. The average POMS scores of the experimental group and the control group after treatment were 6.08 ± 0.43 points and 11.06 ± 0.58 points, respectively; ∗ indicates that the average POMS scores of the two groups of patients after treatment are significantly different (*t* = 46.269, *p* < 0.001).

The POMS score of the experimental group after treatment was lower than that of the control group (*p* < 0.001, Figure 1).

### 3.6. Comparison of GQOLI-74 Scores

**Figure 2:**
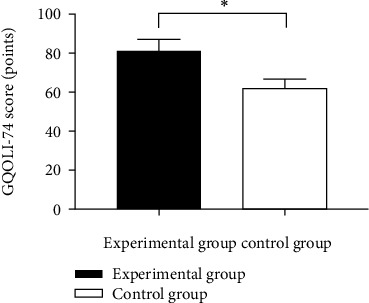
Comparison of GQOLI-74 scores between the two groups ($\overset{¯}{x}\pm s$). Note: the abscissa represents the experimental group and the control group, respectively, and the ordinate represents the GQOLI-74 score and points. The GQOLI-74 scores of the experimental group and the control group after treatment were 81.44 ± 5.72 points and 62.11 ± 4.68 points, respectively; ∗ indicates that there is a significant difference in the GQOLI-74 scores between the two groups of patients after treatment (*t* = 17.545, *p* < 0.001).

The patients receiving tumor plastic surgery showed a remarkably higher GQOLI-74 score versus traditional repair (*p* < 0.001), as shown in Figure 2.

## 4. Discussion

Defects following maxillofacial tumor resection usually involve gums, lips, cheeks and tongues, mandibles, soft tissues of the floor of the mouth, and occlusal relationships [14]. Some patients may have maxillary defects after surgery, which impairs the patient's chewing function, disrupts their language ability, and compromises their quality of life. Mohs' microscopic technique is an important treatment for malignant tumors of the maxillofacial region [5]. Maxillofacial tumors usually invade irregularly into the surrounding tissues. The Mohs' microscopic technique is an effective way to mark and excise tumors while ensuring minimal loss of normal tissue. In recent years, postoperative facial tissue defect repair has become a major challenge faced by plastic surgeons. It is consequently of immense significance to repair facial defects and restore the physiological function of the maxillofacial and facial cosmetic beauty to relieve the physical and mental pressure of the patient [15, 16]. At present, the mainstay of treatment for facial defects after maxillofacial tumors is skin flap repair, yet the choice of the location of the flap remains controversial in spite of its remarkable achievements in the repair of damaged oral function and improvement of the face beauty [17–19]. The pectoralis major myocutaneous flap, with the merits of simple operation and convenient materials, is a thoracic myocutaneous flap pedicled with the thoracic acromion artery. Nevertheless, it is associated with massive damaged donor area, severe postoperative pain, and big gaps between the pectoralis major myocutaneous flap and the maxillofacial area, undermining the aesthetic appearance. The tumor plastic surgery sutures the face according to the skin texture of the patient's facial tissue where the treatment measures are carried out according to the operation of plastic surgery, and targeted and effective measures are performed according to the patient's facial anatomy and the degree of the defect [20, 21]. Direct pulling sutures are suitable for incisions with little tension, while larger defects can be closed directly but are prone to significant postoperative scarring [10]. The skin flap graft requires a separate donor area and high requirements for the survival of the skin flap, and the local flap is formed in the adjacent area of the skin defect, which is suitable for wound repair in the head and face and other areas with high requirements for appearance.

In the present study, the total clinical effective rate and the excellent rate of facial improvement in the experimental group were significantly higher than those in the control group, indicating that the tumor plastic technology can effectively improve the patient's facial defects, mouth opening, and oral occlusal relationship and result in a favorable recovery outcome in maxillofacial physiological function. Similarly, Gómez-Pedraza et al. concluded that rehabilitation of masticatory function improved the patient's systemic and nutritional status, with coverage of the esthetic defect, and these outcomes reduced the psychological and emotional effects of tumor ablation [15]. Previous studies have argued that a failed repair of facial defects during the first-stage treatment leads to leftover hypertrophic scars which would increase the difficulties of the second-stage surgery and impose a substantial economic burden and mental pressure [22, 23]. Heil et al. [24] stated that under the influence of factors such as tension, the scars at the repaired site are more visible with traditional repair, but the alignment outcome is undesirable. It is worth noting that the tumor plastic surgery selects suitable skin flaps according to the actual defect scope and part and uses transplantation to repair the wound, substantially contributing to the recovery of the blood supply of the recipient area. Additionally, it accelerates the healing of surgical injuries while preserving the normal tissues, which fulfills the patient's requirements for facial beauty. The present study reported lower POSAS and POMS scores in the experimental group versus the control group, suggesting that the tumor plastic surgery has an excellent effectiveness profile with respect to the reduction of physical and mental stress and improvement of facial beauty compared with conventional repair techniques. Crown et al. pointed out that oncoplastic breast-conserving surgery (OBCS) has a low rate of significant surgical site complications, with satisfactory cosmetic results [22], which is largely consistent with our study. Moreover, the present study also revealed a higher GQOLI-74 score for the experimental group after treatment versus the control group. Our data are largely consistent with the results from previous research by Péter et al. [25] who also concluded a promising result in the study group (70.18 ± 4.16 vs. 41.24 ± 4.18), further confirming the feasibility of the technique used in the present study. Therefore, the technique used in the observation group may provide clinical benefits for patients in the quality of life. Overall, the present study pioneered the application of tumor plastic technology in maxillofacial tumor resection, which might satisfy patients' aesthetic demands and facilitate rehabilitation. The facial rotation flap is mainly used to select the skin in the preauricular, parotid occlusal, and submandibular areas, which are rich in blood supply. In the present study, a local rotating flap was used for the repair of defects after maxillofacial surgery, i.e., a local flap was formed with the lateral position of the defect and rotated to a certain angle for repair [13]. The flap is rotated to the skin defect line to cover the repaired wound, and the donor area is sutured directly to the flap, which is particularly suitable for round and triangular defects. In addition, other flap designs can be added according to the shape and size of the defect and with reference to the normal surrounding flattening conditions, which is simple and convenient.

## 5. Conclusion

The tumor plastic technology might serve as a promising alternative in maxillofacial tumor resection patients. It considerably improves the patient's quality of life, relieves the patient's psychological pressure, and promotes the rapid recovery of the patient's facial beauty. It merits promotion and application. The limitation of this study is the existence of bias in the included patients. Future studies with large multicenter samples and the acquisition of follow-up data will be conducted to obtain more reliable clinical data.

## Figures and Tables

**Table 1:** Baseline data.

	Experimental group (*n* = 45)	Control group (*n* = 45)	*x* ^2^/*t*	*p*
Gender			0.045	0.832
Male	25 (55.56%)	24 (53.33%)		
Female	20 (44.44%)	21 (35.56%)		
Mean age ($\overset{¯}{x}\pm s$, year)	47.26 ± 3.88	47.52 ± 3.27	0.344	0.732
BMI (kg/m^2^)	21.62 ± 1.05	21.58 ± 1.15		
Tumor type				
Parotid gland cancer	14 (31.11%)	13 (28.89%)	0.053	0.818
Gum cancer	11 (24.44%)	12 (26.67%)	0.058	0.809
Submandibular adenocarcinoma	9 (20.00%)	8 (17.78%)	0.073	0.788
Mouth floor cancer	6 (13.33%)	7 (15.56%)	0.089	0.764
Tongue cancer	5 (11.11%)	5 (11.11%)	0.000	1.000
Staging			0.049	0.824
II	30 (66.67%)	29 (64.44%)		
III	15 (33.33%)	16 (35.56%)		
Educational background				
Elementary and junior high school	2 (4.44%)	3 (6.67%)	0.212	0.645
High school and college	13 (28.89%)	15 (33.33%)	0.207	0.649
University and above	30 (66.67%)	27 (60.00%)	0.431	0.512
Occupation				
Unemployed	4 (8.89%)	5 (11.11%)	0.124	0.725
Worker	5 (11.11%)	4 (8.89%)	0.124	0.725
Farmer	2 (4.44%)	4 (8.89%)	0.714	0.398
Teachers and civil servants	29 (64.44%)	28 (62.22%)	0.048	0.827
Others	5 (11.11%)	4 (8.89%)	0.124	0.725
Places of residence			0.062	0.803
City	10 (22.22%)	11 (24.44%)		
Rural area	35 (77.78%)	34 (75.56%)		

**Table 2:** Clinical efficacy [*n* (%)].

Groups	*n*	Markedly effective	Effective	Ineffective	Total
Experimental group	45	31 (68.89%)	13 (28.89%)	1 (2.22%)	44 (97.79%)
Control group	45	20 (44.44%)	11 (24.44%)	14 (31.11%)	31 (68.89%)
*x* ^2^					13.520
*p*					<0.001

**Table 3:** Facial improvement [*n* (%)].

Groups	*n*	Excellent	Good	Moderate	Poor	Excellent and good rate
Experimental group	45	17 (37.78%)	25 (55.56%)	2 (4.44%)	1 (2.22%)	42 (93.33%)
Control group	45	11 (24.44%)	13 (28.89%)	11 (24.44%)	10 (22.22%)	24 (53.33%)
*x* ^2^						18.409
*p*						<0.001

**(a):** 

Groups	*n*	The degree of pain or itching in the operation area (points)	Color (points)	Hardness (points)	Thickness (points)	Flatness (points)
Experimental group	45	2.55 ± 0.41	2.12 ± 0.32	1.93 ± 0.35	1.15 ± 0.17	1.85 ± 0.27
Control group	45	3.27 ± 0.53	3.37 ± 0.45	3.14 ± 0.66	2.32 ± 0.45	2.33 ± 0.42
*t*		7.208	15.186	10.865	16.316	6.449
*p*		<0.001	<0.001	<0.001	<0.001	<0.001

**(b):** 

Groups	*n*	Degree of hyperemia (points)	Color and lustre (points)	Thickness (points)	Softness (points)	Fitness (points)
Experimental group	45	2.02 ± 0.27	2.01 ± 0.29	1.51 ± 0.21	1.44 ± 0.21	2.15 ± 0.39
Control group	45	2.88 ± 0.42	2.52 ± 0.44	2.08 ± 0.53	2.45 ± 0.38	3.01 ± 0.37
*t*		11.554	6.492	6.707	15.605	10.731
*p*		<0.001	<0.001	<0.001	<0.001	<0.001

## Data Availability

The datasets used during the present study are available from the corresponding author upon reasonable request.
